# Une association d’un goitre ectopique basi-cervical et d’une glande thyroïde eutopique: à propos d’un cas

**DOI:** 10.11604/pamj.2023.45.69.34033

**Published:** 2023-05-31

**Authors:** Ndongo Pilor, Moustapha Ndiaye, Marie Joseph Dieme, Abdoulaye Keïta, Mame Sanou Diouf, Ciré Ndiaye, Abdou Sy, Malick Ndiaye, Evelyne Siga Diom, Abdourahmane Tall, Issa Cheikh Ndiaye

**Affiliations:** 1 Centre Hospitalier National Universitaire de Fann, Université Cheikh Anta Diop, Dakar, Sénégal,; 2 Centre Hospitalier Universitaire d'Aristide Le Dantec, Université Cheikh Anta Diop, Dakar, Sénégal,; 3 Hôpital Général Idrissa Pouye, Université Cheikh Anta Diop, Dakar, Sénégal,; 4 Hôpital d'Enfants de Diamniadio, Université Iba Der Thiam de Thiès, Thiès, Sénégal,; 5 Hôpital de la Paix, Université Assane Seck de Ziguinchor, Ziguinchor, Sénégal

**Keywords:** Glande thyroïde, ectopie, basicervical, cas clinique, Thyroid gland, ectopia, basicervical, case report

## Abstract

La glande thyroïde est une glande endocrine jouant un rôle majeur dans le métabolisme énergétique, phosphocalcique, entre autres. Sa position habituelle est cervicale antérieure et prétrachéale. Les ectopies thyroïdiennes sont des situations assez rares. Elles sont dominées par les ectopies basilinguales. Nous rapportons un cas d'association goitre en position cervicale normale (eutopique) et tissu thyroïdien ectopique en position basicervicale. Le nodule ectopique a été mis en évidence lors de la cervicotomie et le diagnostic confirmé à l'histologie. Ce cas clinique est un diagnostic différentiel des masses cervicales.

## Abstract (en)

Thyroid gland is an endocrine gland playing a major role in energy and phosphocalcic metabolism, among others. It commonly occurs in the anterior cervical and pretracheal region. Ectopic thyroid is quite rare. It is dominated by ectopic basilingual thyroid. We here report the case of a patient with goiter in normal cervical position (eutopic) and ectopic thyroid tissue in basicervical position. Cervicotomy revealed the ectopic nodule and the diagnosis was confirmed by histology. This clinical case illustrates a differential diagnosis of cervical masses.

## Introduction

L'ectopie thyroïdienne est une situation assez rare. Sa prévalence est évaluée à 1/100000-300000 personnes et survient chez 1 patient sur 4000-8000 porteurs d'une pathologie thyroïdienne [1]. Elle correspond à une position atypique de la glande thyroïde en dehors de sa position cervicale pré-trachéale [2]. En effet, lors de la vie fœtale, l'ébauche de la glande thyroïde est en position basi-linguale. Par la suite, une migration descendante vers la région cervicale se fait naturellement. Un défaut de migration explique cette situation d'ectopie [3,4]. Ainsi plusieurs localisations ont été décrites. Les localisations linguales (90%) sont les plus rapportées [1,5]. Les autres localisations médiastinales, cervicales, intra-trachéales et intra-œsophagiennes sont exceptionnelles [4,6]. Le cas rapporté concerne une ectopie thyroïdienne en présence d'une glande thyroïde en position normale (eutopique).

## Patient et observation

**Informations sur le patient:** il s'agit d'une patiente de 42 ans, aux antécédents de césarienne, sans notion de goitre familial, ayant consultée un cardiologue devant l'apparition d'une pesanteur thoracique datant d'un an. Aucune notion de dysphonie ou de dyspnée associée n'avait été rapportée par la patiente. Devant la normalité du bilan cardiopulmonaire (examen clinique et ECG), le cardiologue décida de la réalisation d'une Tomodensitométrie (TDM) et de l'adresser en Oto-rhino-laryngologie (ORL) par la suite.

**Observation clinique:** l'examen retrouvait une masse latéro-trachéale droite basse, ferme et ascensionnant avec la déglutition, sans adénopathies associées. Le reste de l'examen somatique était normal.

**Figure 1:**
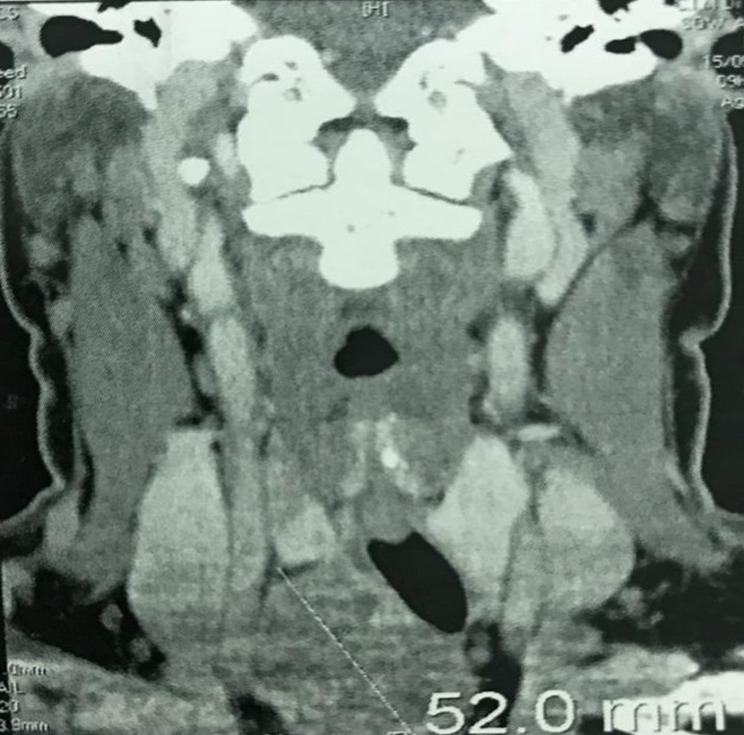
tomodensitométrie (TDM) cervicale: masse latéro trachéale droite, basi-cervicale

**Eléments du diagnostic:** le bilan hormonal thyroïdien était normale (T4: 12,58 pmol/L; T3: 3,91 pmol/L; TSH: 0,703 µUI/mL). L'échographie montrait la présence d'une masse basi-cervicale droite de 5 cm, plongeant et classée TIRADS II. L'examen cytologique après une cytoponction à l'aiguille fine retrouvait des cellules folliculaires bénignes et l'absence de signes de malignité. La TDM demandée par le cardiologue mettait en évidence une masse cervicale de nature tissulaire et plongeante dans le médiastin. La masse était hétérogène, à rehaussement hétérogène et située en zone latéro-trachéale droite et exerçant un effet de masse sur la trachée. Les mensurations étaient de 44X37 mmm dans le plan axial; la masse s'étendait de C6 à T1 (Figure 1).

**Figure 2:**
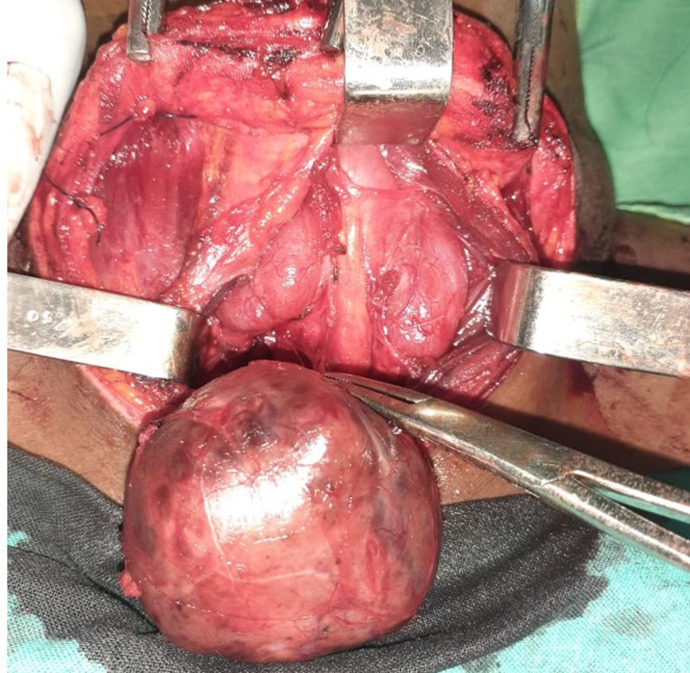
image de cervicotomie

**Intervention thérapeutique:** l'exérèse de la masse avait été effectuée à l'occasion d'une cervicotomie. Il s'agissait d'une masse tissulaire, rosâtre, latéro-trachéale basse droite, plongeant dans le médiastin et adhérant à la trachée par des attaches fibreuses. La dissection de la portion médiastinale s'était faite au doigt. La glande thyroïde était présente et en position normale (Figure 2). Un drain avait été mis en fin d'intervention et retirée 2 jours après

**Figure 3:**
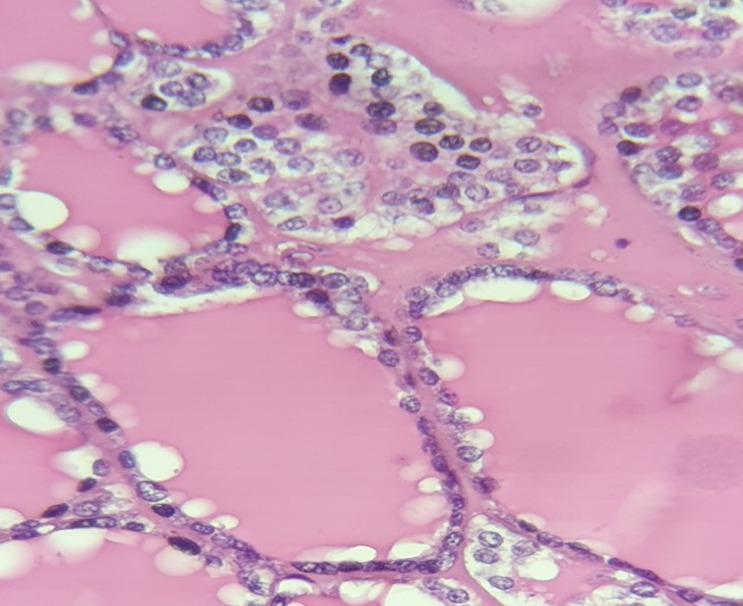
tissu thyroïdien (follicules thyroïdiennes)

**Suivi et résultats:** les suites opératoires étaient simples. L'examen histologique de la pièce opératoire montrait un adénome thyroïdien de type vésiculaire (Figure 3).

**Consentement du patient:** le patient consent à la publication de ses images.

## Discussion

Tout le challenge est de faire la différence entre ectopie avec ou sans glande thyroïde en position normale. A cet égard, deux examens peuvent aider au diagnostic: l'échographie et la scintigraphie [6]. L'échographie permet de vérifier la présence de la glande thyroïde en position cervicale normale alors que la scintigraphie fixe le tissu thyroïdien, permettant ainsi de dépister les localisations les plus atypiques. En l'absence de scintigraphie, comme dans notre cas, une panoplie de diagnostics différentiels s'offre à nous. En cas de localisation cervicale, nous pouvons citer les kystes du tractus thyréoglosses ou les kystes thymiques [7-9].

Les localisations les plus à risque sont intra-trachéale et basi-linguale car pourvoyeurs de dyspnée par obstruction ou d'inondation pharyngo laryngée lors d'un saignement [3,4]. Parfois l'ectopie est asymptomatique ou peut se révéler par des signes trompeurs à type de méno-métrorragies inexpliquées qui doivent être mis dans le compte de l'hypothyroïdie induite [4]. En l'absence d'une glande thyroïde eutopique, un état d'hypothyroïdie est souvent rapporté [3,4,6]. Ainsi la présence d'une glande eutopique et fonctionnelle peut masquer une ectopie thyroïdienne asymptomatique. Dès lors, tout le danger réside dans la possibilité de dégénérescence de la glande ectopique sans signe d'appels évidents [4]. Certains auteurs préfèrent l'abstention thérapeutique en cas d'ectopie asymptomatique [3,4]. En cas de troubles respiratoires par compression, obstruction ou saignement, la chirurgie doit cependant être précipitée.

## Conclusion

L'association goitre ectopique et thyroïde eutopique et fonctionnelle est meilleure sur le plan hormonal thyroïdien après exérèse de la glande ectopique. La scintigraphie doit davantage être effectuée en cas de masse cervicale chronique afin de dépister les goitres ectopiques.
